# Application of nanostructured lipid carriers: the prolonged protective effects for sesamol in in vitro and in vivo models of ischemic stroke via activation of PI3K signalling pathway

**DOI:** 10.1186/s40199-017-0191-z

**Published:** 2017-12-20

**Authors:** Parichehr Hassanzadeh, Fatemeh Atyabi, Rassoul Dinarvand, Ahmad-Reza Dehpour, Morteza Azhdarzadeh, Meshkat Dinarvand

**Affiliations:** 1 Nanotechnology Research Center, Faculty of Pharmacy, Tehran University of Medical Sciences, Tehran, Iran; 2 Department of Pharmaceutics, Faculty of Pharmacy, Tehran University of Medical Sciences, Tehran, Iran; 3 Department of Pharmacology, Faculty of Medicine, Tehran University of Medical Sciences, Tehran, Iran

**Keywords:** Sesamol, Nanostructured lipid carriers, Ischemic stroke, Phosphoinositide 3-kinase, PC12 cells, Rat

## Abstract

**Background:**

Treatment of the ischemic stroke has remained a major healthcare challenge. The phenolic compound, sesamol, has shown promising antioxidant and neuroprotective effects, however, fast clearance may negatively affect its efficiency. This, prompted us to incorporate sesamol into the nanostructured lipid carriers (S-NLCs) and evaluate its therapeutic potential in in vitro and in vivo models of ischemic stroke.

**Methods:**

S-NLCs formulations were prepared by high-pressure homogenization followed by physicochemical characterization, evaluation of the bioactivity of the optimal formulation in oxygen-glucose deprivation (OGD) and global cerebral ischemia/reperfusion (I/R) injury and implication of phosphatidylinositol 3-kinase (PI3K) pathway in this regard. Two- or three-way ANOVA, Mann-Whitney *U* test, and Student’s t-test were used for data analysis.

**Results:**

Formation of S-NLCs which exhibited a controlled release profile, was confirmed by scanning electron microscope and differential scanning calorimetry. 1- and 8-h OGD followed by 24 h re-oxygenation significantly reduced PC12 cell viability, increased lactate dehydrogenase activity and the number of condensed nuclei, and induced oxidative stress as revealed by increased malondialdehyde level and decreased glutathione content and superoxide dismutase and catalase activities. Sesamol (80 and 100 μM) reduced the cytotoxicity, oxidative stress, and cellular damage only after 1-h OGD, while, S-NLCs (containing 80 and 100 μM of sesamol) were effective at both time points. Intravenous injections of S-NLCs (20 and 25 mg/kg) into rats markedly attenuated I/R-induced neurobehavioural deficits, cellular damage, and oxidative stress, while, free sesamol failed. Pre-treatment with PI3K inhibitor, LY294002, abolished the protective effects against OGD or I/R.

**Conclusions:**

S-NLCs improve the pharmacological profile of sesamol and provide longer lasting protective effects for this phenolic phytochemical. This nanoformulation by activating PI3K pathway may serve as a promising candidate for neuroprotection against the cerebral stroke or other neurodegenerative disorders.

**Graphical abstract:**

Sesamol-loaded NLCs, a promising nanoformulation against the ischemic stroke
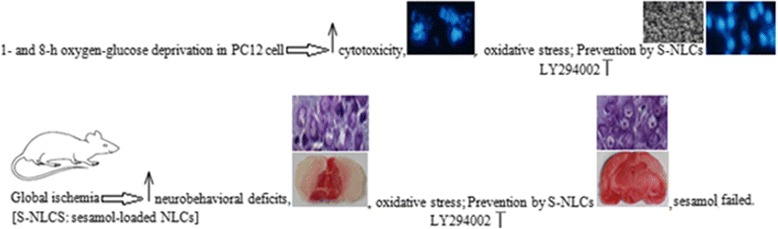

## Background

Ischemic stroke is one of the major causes of disability and death worldwide [1, 2]. Following the severe reduction or interruption of blood flow and oxygen in the cerebral arteries, a sequence of events including the inflammation, oxidative stress, mitochondrial dysfunction, and excessive release of excitatory amino acids may result in the neuronal death [2–4]. Based on the brain’s requirement to a continuous supply of oxygen and glucose, it is the most susceptible organ to the oxygen-glucose deprivation and oxidative stress [5]. The limited efficiency of the currently available medications and their short therapeutic time windows [1], has provoked increasing research efforts to develop novel treatment strategies. During the last decade, antioxidant and neuroprotective effects of the phenolic compounds have been the focus of intense research. In this respect, sesamol, the major constituent of sesame seed oil (*Sesamum indicum*, Linn, Pedaliaceae) has attracted a growing interest due to its high safety profile and wide spectrum of pharmacological activities including the effects against the inflammation, oxidative stress, aging, and depression [6–9]. Moreover, sesamol has shown antithrombotic and neuroprotective properties [10–13] which might be of therapeutic value in the ischemic stroke. However, rapid elimination of sesamol [14, 15] may negatively affect its efficiency that necessitates the development of suitable drug delivery system to improve the stability and bioavailability of this phenolic compound. Over the last few decades, nanotechnology entities have been used to deliver compounds with poor solubility or short half-life [16–23]. There are reports demonstrating the efficiency of nanomaterials for detection or treatment of stroke [24–26]. In recent years, lipid-based colloidal drug delivery systems including the solid lipid nanoparticles (SLNs) and nanostructured lipid carriers (NLCs) as the alternative carrier systems to the liposomes, emulsions, and polymeric nanoparticles have attracted considerable attention. These biocompatible carriers protect the encapsulated active ingredients against the enzymatic degradation and are suitable for targeted drug delivery or controlled release [27]. SLNs have shown advantages of scale-up feasibility, sterility, and protection of incorporated compounds against the degradation, however, the risk of gelation, limited drug-loading capacity, and the possibility of drug leakage during the storage [27–29] led to the development of NLCs, a binary mixture of liquid and solid lipids which provides an imperfect matrix structure. Indeed, NLCs are a smarter generation of drug carriers with high biocompatibility, stability, and drug-loading capacity, prolonged drug residence in the target organ, and minimal drug expulsion during the storage [27, 30, 31]. This background prompted us to prepare sesamol-loaded NLCs (S-NLCs) and evaluate the therapeutic potential of this nanoformulation in both in vitro and in vivo models of ischemic stroke. As a mechanistic approach, we looked at the role of phosphatidylinositol 3-kinase (PI3K) pathway which is critically involved in the cell proliferation and survival [32, 33].

## Methods

### Materials

Cell culture materials were all purchased from GIBCO/Invitrogen, Germany. Cetyl palmitate and Tween 80 were provided by Merck (Darmstadt, Germany) and other chemicals or kits were purchased from Sigma Aldrich, Germany.

### Preparation of sesamol-loaded NLCs (S-NLCs)

S-NLCs were prepared by high-pressure homogenization [34] with some modifications. Briefly, the lipid phase (cetyl palmitate and oleic acid; 85:15 or 70:30) was prepared at 75 °C and sesamol (3,4-methylenedioxyphenol) was added at 5, 10, 20, 40, or 100% *w*/w. The aqueous phase was prepared at 75 °C by dispersing poloxamer 188 (0.5 or 1%, *w*/*v*) and Tween 80 (1 or 2%, w/v) in double-distilled water and was subsequently added to the lipid phase under high-speed stirring (Ultra Turrax T25, IKA, Germany) at 8000 rpm for 30 s. The obtained pre-emulsion was subjected to high-pressure homogenization (Micron LAB 40, Germany) at 500 bar and 75 °C for ten cycles. For further size reduction, the emulsion was sonicated (Ultra sonic, tecno-Gaz Tecna 6, Italy) at 70% amplitude for 2, 4, 10, or 15 min. Afterwards, the nanoemulsion was cooled down to room temperature and then lyophilized (Freeze Drier, Christ, Germany) and stored at 4 °C. Blank NLCs were prepared by the same procedure.

### Characterization of S-NLCs

#### Particle size, polydispersity index (PDI), and zeta potential (ZP)

NLCs dispersions were diluted by deionized water and the mean particle size, PDI, and ZP were analyzed at 25 °C by photon correlation spectroscopy (Zetasizer, Malvern Instruments, UK) (*n* = 6).

### Morphological assessment

Scanning electron microscope (KYKY-EM3200, China) was used to evaluate the shape of nanoparticles.

### Entrapment efficiency (EE) and drug loading capacity (DL)

S-NLC dispersion was placed in the upper chamber of an Amicon® centrifugal filter and centrifuged at 1500 *g* for 30 min. Then, the un-entrapped sesamol in the filtrate was analysed by high-performance liquid chromatography (HPLC) using Alliance 2695 system (Waters Corp, USA) with C18 column (250 × 4.6 mm, 5 μm) at room temperature. The mobile phase contained acetonitrile/water/acetic acid (68:30:2, *v/v*) at flow rate of 1 ml/min and the sample injection volume was 50 μl. The limits of detection and quantification (LOD and LOQ) values were approximately 0.023 and 0.059 μg/ml, respectively, and the inter- and intra-day coefficients of variations were within ±5%. EE% and DL of S-NLCs were determined as follows:

$$ \mathrm{EE}\%=\kern0.5em \frac{\mathrm{Theamount}\  \mathrm{of}\  \mathrm{sesamol}\  \mathrm{encapsulated}\  \mathrm{in}\  \mathrm{NLCs}}{\mathrm{Total}\  \mathrm{amount}\  \mathrm{of}\  \mathrm{sesamol}}\kern0.5em \times \kern0.5em 100 $$

$$ \mathrm{DL}\%=\frac{\mathrm{Theamount}\  \mathrm{of}\  \mathrm{sesamol}\  \mathrm{encapsulated}\  \mathrm{in}\  \mathrm{NLCs}}{\mathrm{Total}\  \mathrm{amount}\  \mathrm{of}\  \mathrm{NLCs}}\times 100 $$

### Differential scanning calorimetry (DSC)

Thermal analysis of pure sesamol, cetyl palmitate, S-NLCs, or blank NLCs was performed by DSC apparatus (Mettler-Toledo, Switzerland). Each sample was placed in an aluminum pan and heated in the range 10–240 °C (10 °C/min) under a nitrogen purge (50 ml/min). Each experiment was carried out in triplicate.

### In vitro release

The release profile of sesamol form NLCs was evaluated by dialysis membrane method [35]. Briefly, 5 ml of S-NLC solution was transferred into a dialysis bag with a molecular weight cut-off of 12 KD (Sigma laboratories, Osterode, Germany) which had been previously soaked in the release medium for 24 h. The dialysis bag was sealed at both ends and immersed in the receptor compartment containing 250 ml of phosphate-buffered saline (PBS, pH 7.4) in a shaking incubator (Heidolph Unimax 1010, Germany) at 37 °C and 100 rpm. At defined time intervals, 0.5-ml samples were collected from the receptor medium, replaced with pre-warmed fresh PBS of an equal volume, and assayed for sesamol content by HPLC. The percentage of dose released was plotted against the time and sesamol solution served as control. Experiments were carried out in triplicate.

### Storage stability

Freeze-dried samples were kept at 4 °C. At defined time intervals (0, 1, 3, and 6 months), samples were re-suspended in filtered water and analyzed for particle size, PDI, ZP, EE %, and DL %. The results were expressed as mean ± SEM (*n* = 6).

### Evaluation of the bioactivity of S-NLCs

#### In vitro experiments

**ᅟ**

**Cell culture**

Rat pheochromocytoma*-*derived PC12 cells were grown in Dulbecco’s modified Eagle’s medium supplemented with fetal bovine serum (10%), horse serum (5%), streptomycin sulfate (100 μg/ml), penicillin G sodium (100 U/ml), and amphotericin B (0.25 μg/ml) in a humidified atmosphere with 5% CO_2_ at 37 °C. The culture media were changed every day and experiments were carried out 72 h after seeding the cells.

#### In vitro model of ischemic stroke and treatments

Oxygen-glucose deprivation (OGD) is a well-recognized in vitro model of stroke and may also be used to develop novel neuroprotective agents or investigate the mechanisms underlying the brain ischemia [36]. PC12 cells were exposed to OGD insult as previously described [37]. In brief, the original culture medium was replaced with pre-warmed glucose-free Hepes buffer {in mM: Hepes (10), NaCl (150), KCl (5), MgCl_2_ (1), CaCl_2_ (2), pH 7.4} and antimycotic-antibiotic solution. Then, the cells were transferred into the anaerobic chamber flushed with 95% N_2_ and 5% CO_2_ for 1 and 8 h at 37 °C. The deoxygenation of media was monitored using the oxygen-sensing probes (dOxyBead™, Luxcel Biosciences, Cork, Ireland). In treatment groups, 20, 40, 80, and 100 μM of sesamol [6–10], S-NLCs (containing 20, 40, 80, and 100 μM of sesamol), vehicle, or blank NLCs were added to the cell cultures 24 h before and upon the OGD onset. In the case of any effect, cells were treated with specific PI3K inhibitor, LY294002, dissolved in 0.2% dimethyl sulfoxide (DMSO) at concentrations of 10, 20, and 50 μM [38, 39] 20 min before the application of the effective agent.

#### Cell viability assay

The viability of PC12 cells was assessed using MTT (3-[4,5-dimethylthiazol-2-yl]-2,5-diphenyl tetrazolium bromide) colorimetric assay [40]. 20 μl of MTT stock solution (5 mg/ml in PBS) was added to each well (1 × 10^4^ cells/well) 4 h before the completion of re-oxygenation period. Then, the culture medium was aspirated and replaced with 100 μl of DMSO and the plate was shaken for 10 min in order to dissolve the crystals and the absorbance was determined at 570 nm (Anthos 2020, Anthos Labtec Instruments, Austria). Cell viability was expressed as the percentage of untreated control cells (assuming the survival rate of 100%) and presented as mean ± SEM of six independent experiments (*n* = 6).

#### Assessment of lactate dehydrogenase (LDH) activity

LDH activity is correlated to the lysed cell numbers [41]. In order to quantify LDH activity, the media were removed following the re-oxygenation period and to obtain the maximum LDH release values, cells were washed twice with PBS and lysed with 1% Triton X-100. The absorbance was recorded at 490 nm and the percentage of LDH release was calculated as follows; [LDH released into the media / (LDH released into the media + LDH released from the lysed cells)] × 100.

#### Morphological analysis

Following the re-oxygentaion period, PC12 cells were cultured in 24-well plates and fixed using 4% paraformaldehyde for 30 min at room temperature. Then, the cells were thoroughly washed with 0.02 M PBS and stained with 10 mg/ml of Hoechst 33,258 in the dark. Images were captured using a fluorescence microscope (Leica, Germany). The condensed nuclei were counted in six visual fields (~ 0.3 mm^2^) in each group and the percentage of condensed nuclei over the total number of cells was calculated as previously described [42].

#### Assessment of the oxidative stress

PC12 cells (5 × 10^5^) were homogenized and centrifuged at 3500 *g* for 20 min at 4 °C and the supernatant was collected for analysis of the reduced glutathione (GSH) and malondialdehyde (MDA) contents and catalase (CAT) and superoxide dismutase (SOD) activities by appropriate kits. Protein content of the supernatant was determined by Bradford method [43]. MDA and GSH contents were determined at 530 and 412 nm, respectively and expressed as nM/mg protein [44, 45]. SOD and CAT activities were evaluated at 560 and 240 nm, respectively and expressed as U/mg protein [46, 47].

#### In vivo experiments

**ᅟ**

**Animals**

Male Wistar rats (300–350 g) from our institution’s laboratory animal centre were randomly assigned and housed under standard laboratory conditions with a 12-h light/dark cycle and food pellets and water provided ad libitum. The protocol for in vivo experiments was approved by the Institutional Animal Care and Use Committee.

#### Visualization of S-NLCs in rat brain

S-NLCs were labeled with coumarin-6 [(3-(2′-benzothiazolyl)-7-diethylaminocoumarin] as previously described [48]. 1 h after the intravenous (i.v.) injection of 25 mg/kg of the labelled S-NLCs, animals were deeply anaesthetized with intraperitoneal (i.p.) injection of ketamine/xylazine, perfused with 0.9% saline and 4% paraformaldehyde, and the brains were stored at −80 °C for 3 days. Afterwards, 20-μm cryosections were provided using a cryostat (Leica, Germany) and were observed under the fluorescence microscope.

#### Brain distribution study

Animals were sacrificed at 0.25, 0.5, 0.75, 1, 2, 4, 6, 8, 10, and 12 h following i.v. injection of 25 mg/kg of S-NLCs or free sesamol and then the brain tissue samples were collected, washed, and weighed for further sesamol analysis [49].

#### Animal groups, induction of ischemic stroke, and treatments

Rats were randomly assigned into the following groups; intact (*n* = 6), sham (*n* = 10), ischemia/reperfusion (I/R), I/R + vehicle, I/R + sesamol, I/R + S-NLCs, LY294002 + I/R + sesamol or S-NLCs, and I/R + blank NLCs (*n* = 14/group). Global cerebral ischemia was induced by a modified four-vessel occlusion (4-VO) method [50]. Animals exhibiting post-ischemic convulsions were excluded from the study. Sham-operated control group underwent the same surgical procedure without carotid ligation. Four days before and immediately after the stroke onset and during the reperfusion period, animals received once-daily i.v. injections of 5, 10, 20, and 25 mg/kg of sesamol (dissolved in 0.5% DMSO) [7–10], S-NLCs [containing the equivalent amounts of sesamol (5, 10, 20, and 25 mg/kg)], vehicle, or blank NLCs. In the case of any effect, LY294002 was dissolved in 3% DMSO and administered intracerebroventricularly (i.c.v.) at concentrations of 5, 10, and 25 μg/μl [51, 52] 20 min prior to the application of the effective agent.

#### Neurological deficit scoring

Post-ischemic neurological deficits were scored on a 5-point scale by an experimenter blinded to the treatment groups as follows, 0: no deficits, 1: difficulty in fully extending the contralateral forelimb, 2: unable to extend the contralateral forelimb, 3: mild circling to the contralateral side, 4: severe circling, and 5: falling to the contralateral side [53].

#### Behavioural assessments

Spatial learning and memory was assessed by Morris water-maze (MWM) test as previously described [54]. In order to assess the emotional memory, step-through passive avoidance test (STPAT) was performed [12].

#### Assessment of the brain infarcted area

Rats were anesthetized with 3.5% chloral hydrate and subjected to the intracardiac perfusion with 100 ml of isotonic saline. Then, animals were killed by decapitation and the brains were removed and sliced coronally at 2-mm intervals. Following the removal of dura mater and vascular tissue, slices were immersed into 2% 2,3,5-triphenyltetrazolium chloride (TTC, Sigma-Aldrich, Germany) solution in PBS (pH 7.4) for 30 min at 37 °C. Slices were turned over for several times and then washed twice in 10 ml of saline and kept in 10% phosphate buffered formalin (pH 7.4) overnight in a lightproof container for further photography. Infarcted areas of the brain sections were integrated and expressed as the percentage of total area [55].

#### Histological assessments

Neuronal loss in the hippocampal CA1 region was assessed as previously described [56]. Following the reperfusion period, animals were anesthetized with chloral hydrate (400 mg/kg, i.p.) and perfused intracardially with heparinized saline followed by 4% paraformaldehyde in 0.1 M PBS (pH 7.4). The brains were carefully removed, post-fixed in 4% paraformaldehyde for 24 h at 4 °C, and embedded in paraffin. For histological assessments which were performed by two observers blind to the experimental set-up, 5 μm-thick coronal sections from the dorsal hippocampus were prepared by a rotary microtome and stained with cresyl violet. Images were captured using a microscope (Olympus Optical Co, LTD, Japan) equipped with a digital camera system (Pixera 600CL-CU, Pixera Corporation, Japan). The survived neurons in CA1 regions of hippocampi were counted in 6 frames (1 mm^2^/each) in five coronal sections and expressed as the percentage of total cell number.

#### Biochemical analysis

In other groups of animals, brains were removed and the hippocampi were immediately dissected on a frozen pad taken from a − 80 °C freezer [57], weighed, homogenized in ice-cold 0.1 M phosphate buffer (pH 7.4), sonicated for 30 s, and centrifuged at 12,000 *g* for 10 min. Then, the supernatants were collected to assess MDA and GSH contents and SOD and CAT activities as aforementioned. The protein contents of the supernatants were determined by Bradford method [43].

#### Statistical analysis

The normal distribution of data was verified by Shapiro-Wilk test. Two- or three-way ANOVA followed by Tukey’s post hoc test were applied to analyse the cytotoxicity, behavioural, and biochemical data. Mann-Whitney *U* test was used to compare the neuron counts, brain infarcted area, and neurological deficit scores. Student’s t-test was used in brain distribution study. Data are presented as mean ± SEM and the statistical significance was set at *P* < 0.05.

## Results

### Characterization of S-NLCs

**Fig. 1:**
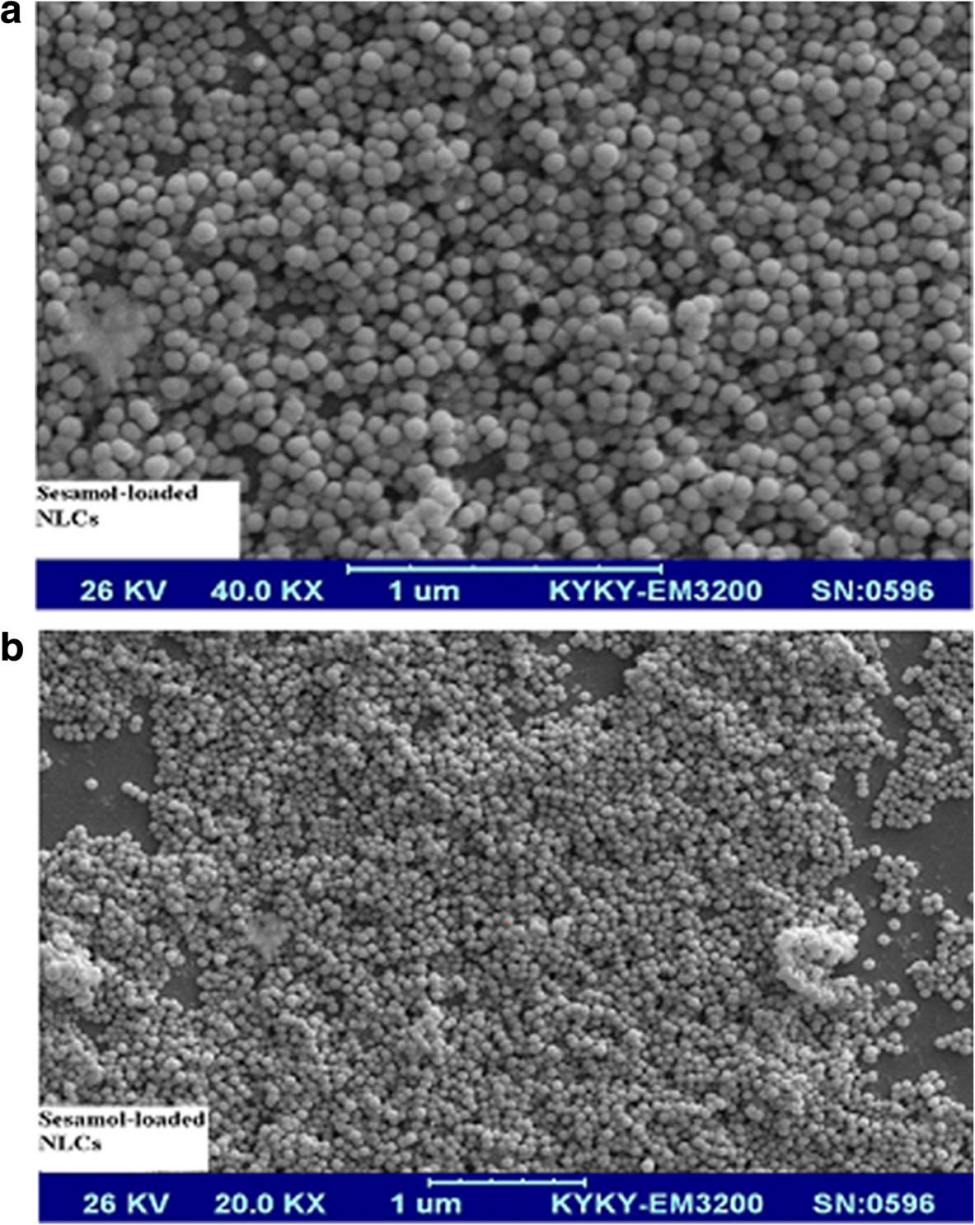
Scanning electron micrographs of sesamol-loaded NLCs. The spherical shape and smooth surface with relatively uniform size are observed in highly-concentrated nanoparticles (formulation S-NLC7). **a** fresh sample with higher magnification, no pore on the nanoparticle surface is observed, **b** The non-aggregated and finely-dispersed nanoparticles three months after freeze drying

**Fig. 2:**
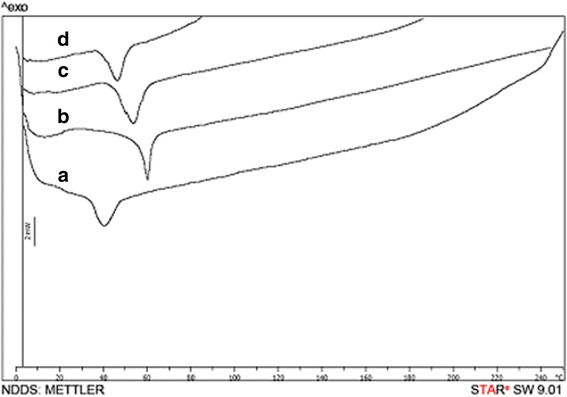
The overlaid DSC thermograms. **a**: sesamol-loaded NLCs three months after freeze drying (formulation S-NLC7), **b**: sesamol, **c**: cetyl palmitate, **d**: blank NLCs. (DSC: differential scanning calorimetry)

**Fig. 3:**
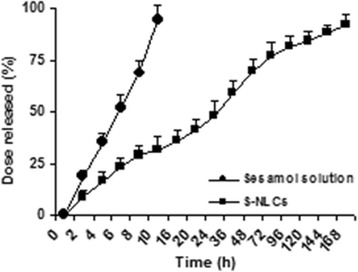
In vitro release profile of sesamol. A controlled release pattern of sesamol from S-NLCs is observed. Data are expressed as the mean ± SEM (*n* = 3). (S-NLCs: sesamol-loaded nanostructured lipid carriers)

S-NLCs dispersions were successfully prepared by a modified high-pressure homogenization technique without signs of phase separation or colour change during at least 1-month visual inspection. Using different ratios of solid and liquid lipids, sesamol and lipid, and surfactants or sonication time, various S-NLCs formulations with different physicochemical properties were prepared. Application of the higher amount of oil lipid or surfactant resulted in the smaller particle size and higher entrapment efficiency (Table 1). Considering the EE%, DL%, and particle size, S-NLCs7 (Table 1) was selected as the optimal formulation for further experimental procedures. Based on the representative SEM images, the freshly-prepared S-NLCs (Fig. 1a) or those three months after freeze-drying (Fig. 1b) were spherical in shape and uniform in size. In DSC thermograms, pure sesamol showed a sharp endothermic peak around 64.59 °C (Fig. 2b) and the melting peak of cetyl palmitate was observed at 54.86 °C (Fig. 2c). The blank NLCs showed melting peak at 47.63 °C (Fig. 2d), while, S-NLCs displayed a melting peak at 43.81 °C three months after freeze drying (Fig. 2a). The release of sesamol from the solution was faster than S-NLCs formulation in which a controlled release pattern was observed (Fig. 3). In various S-NLCs formulations, the total release of 52.7 ± 4.8 to 77.8 ± 6.2% was achieved at the end of 48 h (Table 1). As shown in Table 2, the lyophilized nanoparticles remained stable at 4 °C without significant alterations in the particle size, PDI, ZP, EE or DL % during 6 months of storage (*P* > 0.05).

**Table 1:** Physichochemical properties of sesamol-loaded NLCs

Formulation code	Particle size (nm)	PDI	ZP (mV)	EE (%)	DL (%)	DR after 48 h (%)
S-NLC1 S-NLC2 S-NLC3 S-NLC4 S-NLC5 S-NLC6 S-NLC7 S-NLC8 Blank NLCs	137.4 ± 9.2 123.6 ± 7.4 104.3 ± 8.7 66.3 ± 1.6 73.8 ± 4.3 81.6 ± 3.7 92.3 ± 6.2 124.7 ± 9.4 52.4 ± 6.7	0.37 ± 0.04 0.32 ± 0.01 0.28 ± 0.03 0.12 ± 0.07 0.18 ± 0.07 0.21 ± 0.05 0.23 ± 0.04 0.29 ± 0.09 0.13 ± 0.06	−21.8 ± 0.51 −26.2 ± 0.37 −23.3 ± 0.63 −25.8 ± 0.58 −21.4 ± 0.47 −19.5 ± 0.53 −27.9 ± 0.56 −25.4 ± 0.45 −18.7 ± 0.72	41.4 ± 2.6 47.7 ± 3.2 66.3 ± 8.5 94.3 ± 3.7 92.7 ± 4.3 89.2 ± 6.4 94.2 ± 1.8 91.4 ± 2.5 _	1.45 ± 0.14 1.63 ± 0.08 1.78 ± 0.12 3.11 ± 0.06 6.13 ± 0.17 11.32 ± 3.4 28.51 ± 1.2 49.92 ± 3.7 _	52.7 ± 4.8 57.3 ± 5.3 68.5 ± 4.7 82.3 ± 5.7 77.8 ± 6.2 74.6 ± 3.9 69.8 ± 5.4 60.3 ± 1.8 _

Data are represented as mean ± SEM (*n* = 6)

(*PDI* polydispersity index, *ZP* zeta potential, *EE* entrapment efficiency, *DL* drug loading, *DR* drug release, *S-NLCs* sesamol-loaded nanostructured lipid carriers)

**Table 2:** The stability profile of sesamol-loaded NLCs

Initial					1st Month				
Size	PDI	ZP	EE%	DL%	Size	PDI	ZP	EE%	DL%
92.3 ± 6.2 ± 1.2	0.23 ± 0.04	−27.9 ± 0.5	94.2 ± 1.8	28.51	95.1 ± 4.9	0.19 ± 0.09	−25.3 ± 0.3	92.7 ± 5.4	26.84 ± 1.7
3rd Month					6th Month				
Size	PDI	ZP	EE%	DL%	Size	PDI	ZP	EE%	DL%
94.3 ± 4.1	0.21 ± 0.07	−26.5 ± 0.4	95.5 ± 2.4	28.03 ± 1.9	99.8 ± 3.3	0.19 ± 0.07	−23.7 ± 0.8	89.62 ± 4.7	25.86 ± 0.9

Data are represented as mean ± SEM (n = 6)

(*PDI* polydispersity index, *ZP* zeta potential, *EE* entrapment efficiency, *DL* drug loading, *S-NLCs* sesamol-loaded nanostructured lipid carriers)

### Evaluation of the bioactivity of S-NLCs

#### MTT assay

**Fig. 4:**
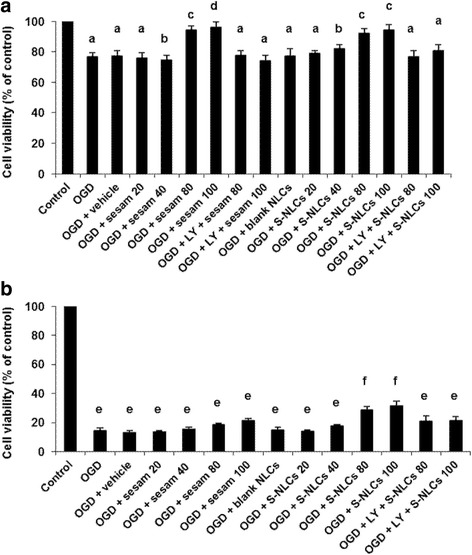
Viability assay in PC12 cell culture. Following 1- and 8-h OGD insult (**a** and **b**, respectively), the viability of PC12 cells were assessed by MTT assay. As shown, S-NLCs elevate the cell viability for longer time period. Pre-treatment with LY294002 (50 μM) reversed the cytoprotective effects. Data are presented as mean ± SEM of six independent experiments. (OGD: oxygen-glucose deprivation, OGD + sesam 20, 40, 80, and 100: administration of 20–100 μM of sesamol 24 h before and upon the OGD insult, OGD + S-NLCs 20, 40, 80, and 100: administration of S-NLCs **(**containing 20–100 μM of sesamol) 24 h before and upon the OGD insult, LY: LY294002, S-NLCs: sesamol-loaded nanostructured lipid carriers). ^a, e^ P < 0.001 and ^b^ P < 0.01 vs. the control group, ^c^ P < 0.05 and ^d^ P < 0.01 vs. OGD and OGD + vehicle, ^f^ P < 0.001 vs. control, OGD, and OGD + vehicle

Following 1- and 8-h OGD insult, cell viability was significantly decreased (Fig. 4, *P* < 0.001 vs. control). Sesamol (80 and 100 μM) prevented the cell loss after 1-h OGD/R (Fig. 4a, P<0.05, *P* < 0.01 vs. OGD or OGD + vehicle group), while, it was ineffective after 8-h OGD/R (Fig. 4b, *P*>0.05). S-NLCs (containing 80 and 100 μM of sesamol) significantly prevented the cell loss after 1- and 8-h OGD/R (Fig. 4a and b, *P*<0.05, *P* < 0.001). Pre-treatment with LY294002 (50 μM) prevented the cytoprotective effects of sesamol or S-NLCs (Figs 4a and b, *P*>0.05 vs. OGD or OGD + vehicle group). Pre-treatment with lower doses of LY294002 showed no effect and this antagonist had no effect by itself (data not shown). Sesamol or S-NLCs (20, 40, 80, and 100 μM) did not induce cytotoxicity in PC12 cells under the normal condition (not shown).

#### LDH release

**Fig. 5:**
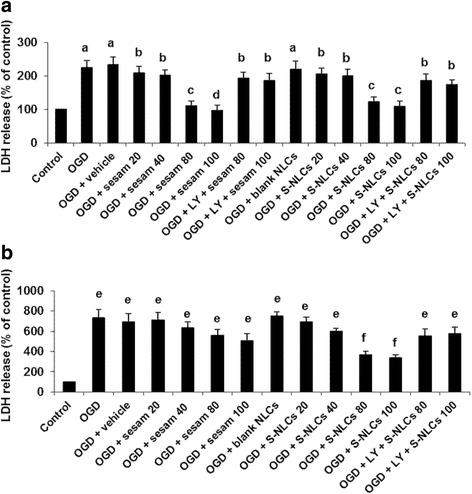
The effect of OGD, sesamol, or S-NLCs on LDH release. S-NLCs effectively prevented the enhancement of LDH release after both 1-h (**a**) and 8-h OGD (**b**) that was reversed due to the pre-treatment with LY294002 (50 μM). Data are presented as the mean ± SEM of six independent experiments. ^a, e^ P < 0.001, and ^b^ P < 0.01 vs. the control, ^c, d^ P < 0.01 vs. OGD and OGD+ vehicle, ^f^ P < 0.05 vs. control and P < 0.001 vs. OGD and OGD + vehicle. (LDH: lactate dehydrogenase, OGD: oxygen-glucose deprivation, LY: LY294002, S-NLCs: sesamol-loaded nanostructured lipid carriers)

LDH release was significantly increased in PC12 cells exposed to 1- or 8-h OGD/R (Fig. 5, *P* < 0.001 vs. control). Sesamol or S-NLCs (80 and 100 μM) prevented the enhancement of LDH activity due to 1-h OGD/R (Fig. 5a, P<0.01 and P < 0.001 vs. OGD or OGD + vehicle group). S-NLCs, but not sesamol, reduced LDH release following 8-h OGD/R (Fig. 5b, *P*<0.05 vs. OGD or OGD + vehicle group). Pre-treatment with LY294002 (50 μM) reversed the effects of sesamol or S-NLCs (Fig. 5a and b, P>0.05 vs. OGD or OGD + vehicle group), while, the lower doses of LY294002 showed no effect (data not shown). LY294002 showed no effect per se (not shown). Sesamol or S-NLCs (20, 40, 80, and 100 μM) did not significantly affect LDH release form PC12 cells under the normal condition (not shown).

#### Morphological alterations in PC12 cells induced by OGD

**Fig. 6:**
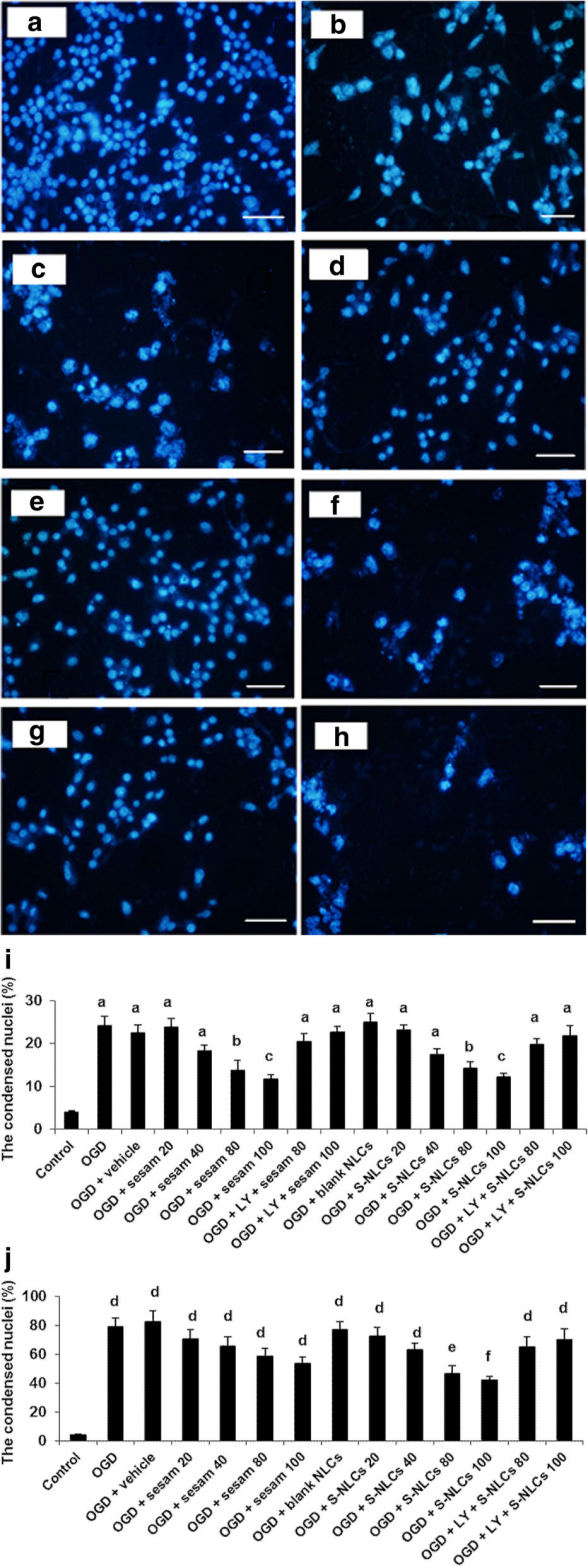
Morphological alterations in PC12 cells exposed to OGD and quantitative analysis of the condensed nuclei. A high density of round cells is demonstrated in the control cells, while, alteration in the cellular morphology and enhancement of condensed nuclei occurred following 1- and 8-h OGD. Treatment of PC12 cells with sesamol or S-NLCs 24 h before and upon the exposure to 1-h OGD resulted in the ameliorative effects. Unlike free sesamol, S-NLCs preserved the protective effects following the longer exposure to OGD insult. Pre-treatment with LY294002 (50 μM) reversed the cytoprotective effects. **a**: control cells, **b**: 1-h OGD insult, **c**: 8-h OGD insult, **c**: sesamol (100 μM) + 1-h OGD, **e**: S-NLCs (100 μM) + 1-h OGD, **f**: sesamol (100 μM) + 8-h OGD, **g**: S-NLCs (100 μM) + 8-h OGD, **h**: LY294002 (50 μM) + S-NLCs (100 μM) + 8-h OGD (scale bars: 50 μm), **i**: (1-h OGD insult), **j** (8-h OGD insult). The numbers of condensed nuclei have been represented as the percentage of total number of nuclei counted. Data are mean ± SEM of six different cell culture preparations. ^a, d^ P < 0.001 vs. the control group, ^b^ P < 0.01 vs. control, OGD, and OGD + vehicle,^c^ P < 0.05 vs. control, and P < 0.001 vs. OGD and OGD + vehicle, ^e^ P < 0.001 vs. control and P < 0.01 vs. OGD and OGD + vehicle, ^f^ P < 0.001 vs. control, OGD, and OGD + vehicle. (OGD: oxygen-glucose deprivation, LY: LY294002, S-NLCs: sesamol-loaded nanostructured lipid carriers)

Exposure to 1- or 8-h OGD/R resulted in the reduced cell number and alteration in cellular morphology (Fig. 6b and c, respectively) as compared to the control (Fig. 6a). Sesamol (100 μM) attenuated the cellular damage due to 1-h OGD (Fig. 6d), however, it was ineffective following 8-h OGD insult (Fig. 6f). Cells treated with S-NLCs (containing 100 μM of sesamol) displayed improved morphology following both 1- and 8-h OGD (Fig. 6e and g, respectively). Pretreatment with LY294002 (50 μM) prevented the cytoprotective effect of S-NLCs (Fig. 6h). The significant enhancement of condensed nuclei induced by 1- or 8-h OGD (Fig. 6i and j, *P*<0.001 vs. the control) was prevented by 80 and 100 μM of sesamol or S-NLCs after 1-h OGD (Fig. 6i, *P*<0.01, *P* < 0.001). S-NLCs (containing 80 and 100 μM of sesamol), but not free sesamol, prevented the formation of condensed nuclei due to 8-h OGD insult (Fig. 6j, *P*<0.001). Pretreatment with LY294002 (50 μM) prevented the cytoprotective effects (Fig. 6i and j, *P*>0.05 vs. OGD or OGD + vehicle group), however, the lower doses of this antagonist were ineffective (not shown). LY294002 showed no effect by itself (not shown).

#### The effects of sesamol or S-NLCs on OGD-induced oxidative stress

As shown in Table 3, induction of OGD for 1 or 8 h resulted in a significant elevation of MDA level and reduction of GSH content and CAT and SOD activities in PC12 cells (*P* < 0.001 vs. control). Following 1-h OGD, 80 and 100 μM of free sesamol reduced MDA (*P* < 0.05 and *P* < 0.01 vs. OGD and OGD + vehicle groups), and elevated GSH content (*P* < 0.05) and activities of SOD (*P* < 0.05, *P* < 0.01) and CAT (*P* < 0.001), however, sesamol was ineffective following 8-h OGD (*P* > 0.05). S-NLCs (containing 80 and 100 μM of sesamol) showed antioxidant effects after both 1- and 8-h OGD (MDA: *P* < 0.01, *P* < 0.001; GSH: *P* < 0.05; SOD: *P* < 0.05, *P* < 0.001; and CAT: P < 0.001 vs. OGD or OGD + vehicle group). Pre-treatment with LY294002 (50 μM) prevented the antioxidant effects (*P* > 0.05 vs. OGD or OGD + vehicle group), while the lower doses were ineffective (not shown). LY294002 showed no effect by itself (not shown).

**Table 3:** Effects of sesamol or S-NLCs on the biochemical parameters following the ischemic insult

Groups	MDA (nM/mg protein)		GSH (nM/mg protein)		SOD (U/mg protein)		CAT (U/mg protein)	
	1 h	8 h	1 h	8 h	1 h	8 h	1 h	8 h
Control OGD OGD + vehicle OGD + sesamol (20 μM) OGD + sesamol (40 μM) OGD + sesamol (80 μM) OGD + sesamol (100 μM) LY + OGD + sesamol (80 μM) LY + OGD + sesamol (100 μM) OGD + blank NLCs OGD + S-NLCs (20 μM) OGD + S-NLCs (40 μM) OGD + S-NLCs (80 μM) OGD + S-NLCs (100 μM) LY + OGD + S-NLCs (80 μM) LY + OGD + S-NLCs (100 μM)	0.53 ± 0.04 1.32 ± 0.10^**^ 1.37 ± 0.14^**^ 1.38 ± 0.23^**^ 1.22 ± 0.19^**^ 0.65 ± 0.06^&^ 0.59 ± 0.03^π^ 1.43 ± 0.09^**^ 1.28 ± 0.15^**^ 1.35 ± 0.16^**^ 1.15 ± 0.11^*^ 1.17 ± 0.08^*^ 0.58 ± 0.03^¥^ 0.47 ± 0.04^∑^ 1.23 ± 0.07^*^ 1.37 ± 0.19^**^	0.57 ± 0.05 2.02 ±0.25^***^ 1.91 ±0.14^***^ 2.12 ±0.21^***^ 1.76 ±0.13^***^ 1.88 ±0.11^***^ 1.79 ±0.09^***^ 1.94 ±0.22^**^ 1.67 ±0.19^**^ 2.07 ±0.17^***^ 1.98 ±0.10^***^ 1.92 ±0.16^***^ 1.02 ±0.16^Ө^ 0.92 ±0.07^∞^ 1.48 ±0.33^**^ 1.27 ±0.08^***^	12.97 ± 1.19 7.13 ± 0.49^***^ 7.29 ± 0.57^***^ 7.92 ± 0.48^***^ 7.22 ± 0.45^***^ 10.62 ± 0.83^#^ 10.29 ± 0.52^∆^ 7.56 ± 0.62^**^ 8.19 ± 0.62^**^ 7.58 ± 0.67^***^ 7.38 ± 0.81^***^ 8.25 ± 0.89^**^ 10.87 ± 0.55^$^ 11.03 ± 0.42© 8.07 ± 0.24^***^ 7.58 ± 0.13^***^	12.71 ± 0.93 3.81 ± 0.36^***^ 3.65 ±0.22^***^ 3.58 ± 0.17^***^ 3.81 ± 0.25^***^ 4.83 ±0.28^***^ 4.17 ±0.13^***^ 4.33 ±0.24^***^ 4.61 ±0.17^***^ 3.47 ±0.18^***^ 4.26 ± 0.39^***^ 4.75 ±0.17^***^ 6.67 ±0.59^@^ 7.49 ± 0.38^£^ 4.25 ±0.19^***^ 4.72 ±0.22^***^	39.67 ± 2.94 24.94 ± 2.38^***^ 23.63 ± 1.82^***^ 23.02 ± 2.11^***^ 26.83 ± 1.96^**^ 37.06 ± 1.89^ω^ 36.94 ± 2.14^Ω^ 25.17 ± 1.03^***^ 23.86 ± 2.87^**^ 25.17 ± 2.34^***^ 27.70 ± 1.94^**^ 29.38 ± 1.33^*^ 35.33 ± 2.12^€^ 34.52 ± 1.84^Ϙ^ 24.13 ± 1.08^**^ 22.96 ± 1.85^***^	38.29 ± 1.87 18.77 ± 1.14^***^ 19.35 ± 1.29^***^ 19.83 ± 1.82^***^ 18.22 ± 2.16^***^ 21.63 ± 1.96^***^ 20.17 ± 1.07^***^ 18.73 ± 2.45^***^ 21.08 ± 1.66^***^ 18.16 ± 1.01^***^ 17.38 ± 1.64^***^ 20.52 ± 2.02^***^ 27.84 ± 2.14^ø^ 29.03 ± 1.23^ж^ 17.88 ± 1.13^***^ 21.32 ± 1.89^***^	6.83 ± 0.44 2.44 ± 0.19^***^ 2.32 ± 0.25^***^ 2.45 ± 0.14^***^ 2.76 ± 0.27^***^ 4.28 ± 0.36^Φ^ 4.86 ± 0.19^Ҩ^ 2.34 ± 0.23^**^ 2.69 ± 0.14^***^ 2.36 ± 0.15^***^ 2.23 ± 0.15^***^ 3.42 ± 0.24^***^ 4.86 ± 0.27 ^β^ 4.73 ± 0.14^ȹ^ 3.11 ± 0.19^***^ 2.65 ± 0.27^**^	6.88 ± 0.47 1.49 ± 0.18^***^ 1.53 ± 0.16^***^ 1.47 ± 0.13^***^ 1.64 ± 0.11^***^ 1.59 ± 0.16^***^ 1.73 ± 0.08^***^ 1.29 ± 0.18^**^ 1.55 ± 0.07^***^ 1.54 ± 0.12^***^ 1.71 ± 0.18^***^ 1.85 ± 0.21^***^ 2.83 ± 0.26^Џ^ 2.96 ± 0.23^ʉ^ 1.63 ± 0.09^***^ 1.96 ± 0.16^***^

PC12 cells have been exposed to 1 and 8 h oxygen-glucose deprivation. Each value represents the mean ± SEM of six independent experiments

(*MDA* malondialdehyde, *GSH* reduced glutathione, *CAT* catalase, *SOD* superoxide dismutase, *OGD* oxygen-glucose deprivation, *S-NLCs* sesamol-loaded nanostructured lipid carriers, *LY* LY294002)

^*^ *P* < 0.05, ^**^ *P* < 0.01, ^***^ *P* < 0.001 vs. control, ^#, $,^ ©^, &, €, Ϙ, ∆, @, £, Ω^ *P* < 0.05 vs. OGD and OGD + vehicle, ^π, ¥, ∑, Ө, ω^P < 0.01 vs. OGD and OGD + vehicle, ^∞, Φ, Ҩ, β, ȹ, ж^ *P* < 0.001 vs. control, OGD, and OGD + vehicle, ^ø^ P < 0.01 vs. control and P < 0.05 vs. OGD and OGD + vehicle, ^Џ, ʉ^ P < 0.001 vs. control and P < 0.01 vs. OGD and OGD + vehicle

#### In vivo experiments

**ᅟ**

**Brain delivery of S-NLCs**

**Fig. 7:**
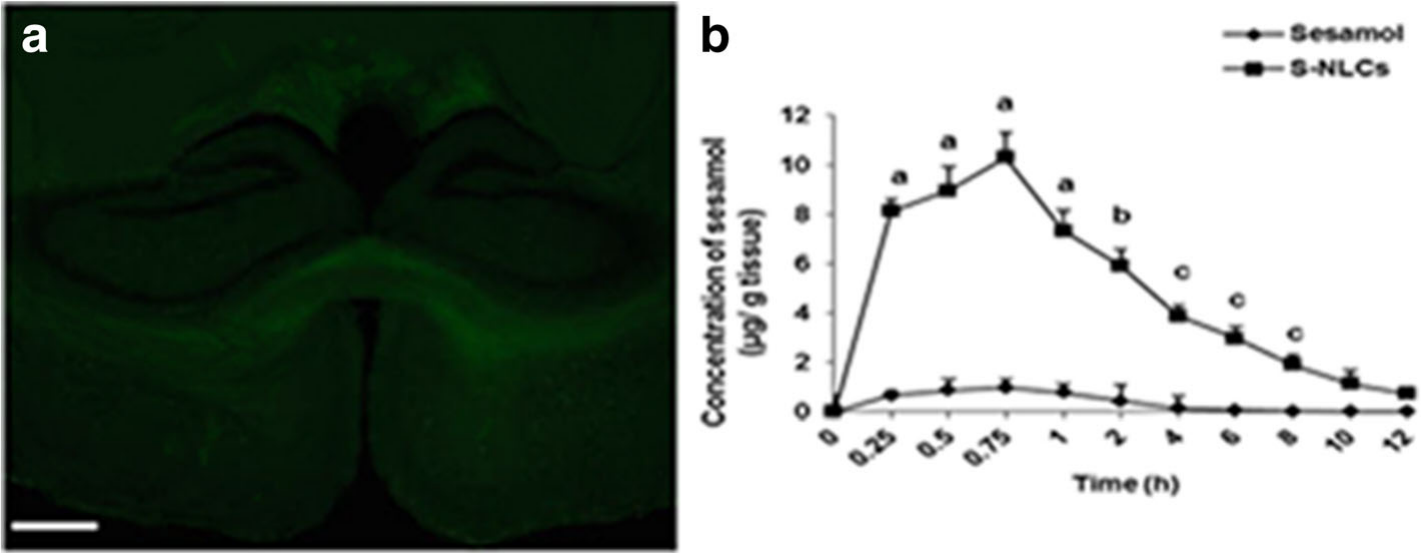
The brain delivery of S-NLCs. **a**: The presence of coumarin-6-loaded S-NLCs in the hippocampus which is one of the most vulnerable brain regions in the ischemic stroke (scale bar: 500 μm), **b**: Brain distribution study shows that S-NLCs provide significantly higher concentrations of sesamol in the brain. ^a^ P < 0.001, ^b^ P < 0.01, and ^c^ P < 0.05 vs. sesamol-treated group. (S-NLCs: sesamol-loaded nanostructured lipid carriers)

Using coumarin-6-loaded nanoparticles, S-NLCs entry into the hippocampus was demonstrated (Fig. 7a). Based on the brain distribution study, administration of S-NLCs provided a significantly higher brain concentrations of sesamol as compared to the free sesamol (Fig. 7b, *P*<0.001).

#### The effects of sesamol solution or S-NLCs on I/R-induced neurobehavioral deficits

As shown Table 4, neurological deficit score was significantly increased in I/R or I/R + vehicle group (*P <* 0.001 vs. sham or intact group) and decreased in the groups treated with S-NLCs containing 20 or 25 mg/kg of sesamol (*P* < 0.01 and *P* < 0.001 vs. I/R or I/R + vehicle group). Furthermore, global ischemia impaired the learning and memory as revealed by MWMT or STPAT (*P* < 0.001 vs. sham or intact group). Treatment with S-NLCs (20 and 25 mg/kg) significantly improved the learning and memory (*P* < 0.05 and *P* < 0.001 vs. I/R or I/R + vehicle group). The free sesamol failed to affect I/R-induced neurobehavioral deficits at any dose tested (*P* > 0.05 vs. I/R or I/R + vehicle group). Pretreatment with LY294002 (25 μg/μl) abolished the ameliorative effects of S-NLCs (*P* > 0.05 vs. I/R or I/R + vehicle group). Pretreatment with lower doses of LY294002 showed no effect (not shown). LY294002 had no effect by itself (not shown).

**Table 4:** The effects of sesamol or S-NLCs on I/R-induced neurobehavioral deficits

Groups scores	Neurological (1–5)	MWMT TSTQ (s)	STPAT Latency (s)
Sham Intact I/R I/R + vehicle I/R + sesamol (5 mg/kg) I/R + sesamol (10 mg/kg) I/R + sesamol (20 mg/kg) I/R + sesamol (25 mg/kg) I/R + blank NLCs I/R + S-NLCs (5 mg/kg) I/R + S-NLCs (10 mg/kg) I/R + S-NLCs (20 mg/kg) I/R + S-NLCs (25 mg/kg) LY + I/R + S-NLCs (20 mg/kg) LY + I/R + S-NLCs (25 mg/kg)	0 0 3.84 ± 0.36 ^a^ 3.67 ± 0.44 ^a^ 3.44 ± 0.49 ^a^ 3.52 ± 0.23 ^a^ 3.74 ± 0.38 ^a^ 2.98 ± 0.29 ^a^ 4.03 ± 0.26 ^a^ 3.57 ± 0.22 ^a^ 3.28 ± 0.17 ^a^ 1.76 ± 0.13 ^b^ 1.27 ± 0.07 ^c^ 3.55 ± 0.47 ^a^ 3.21 ± 0.29 ^a^	75.38 ± 6.26 77.21 ± 4.23 16.33 ± 1.14 ^a^ 14.83 ± 0.79 ^a^ 17.66 ± 1.02 ^a^ 15.79 ± 0.76 ^a^ 19.49 ± 1.31 ^a^ 16.65 ± 1.27 ^a^ 18.33 ± 0.62 ^a^ 26.42 ± 2.41 ^a^ 23.79 ± 2.29 ^a^ 59.61 ± 3.76 ^d^ 66.27 ± 4.33^c^ 18.43 ± 1.75 ^a^ 16.77 ± 2.93 ^a^	283.17 ± 10.69 278.42 ± 12.18 179.11 ± 9.46 ^a^ 184.40 ± 11.32 ^a^ 176.61 ± 7.47 ^a^ 185.63 ± 11.33 ^a^ 183.57 ± 9.97 ^a^ 195.46 ± 12.68 ^a^ 173.77 ± 7.63 ^a^ 189.27 ± 16.54 ^a^ 177.11 ± 11.13 ^a^ 217.50 ± 9.56 ^e^ 237.33 ± 13.10 ^c^ 180.16 ± 9.76 ^a^ 191.45 ± 12.37 ^a^

S-NLCs, but not free sesamol, significantly attenuated the neurobehavioral deficits induced by global ischemia. Data are presented as mean ± SEM (*n* = 6)

(*I/R* ischemia/reperfusion, *S-NLCs* sesamol-loaded nanostructured lipid carriers, *LY* LY294002 (25 μg/μl), *MWMT* Morris water maze test, *TSTQ* time spent in target quadrant, *STPAT*: step-through passive avoidance test, latency; time spent in crossing from the illuminated to the darkened compartment)

^a^ *P* < 0.001 vs. sham or intact, ^b^ *P* < 0.01 vs. sham, intact, I/R, or I/R + vehicle, ^c^ P < 0.001 vs. I/R or I/R + vehicle, ^d^ P < 0.01 vs. sham or intact and P < 0.001 vs. I/R or I/R + vehicle, ^e^ *P* < 0.05 vs. I/R or I/R + vehicle

#### The effects of S-NLCs or sesamol on the brain infarction induced by global cerebral I/R

**Fig. 8:**
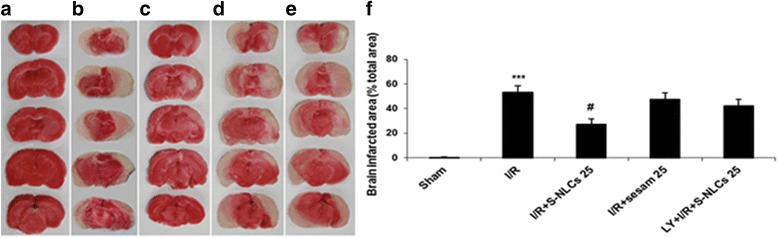
Representative photographs of brain coronal sections stained with TTC. **a**: sham, **b**: I/R (infarcted areas have been shown in white), **c**: S-NLCs (containing 25 mg/kg of sesamol) + I/R, **d**: sesamol (25 mg/kg) + I/R, **e**: LY294002 (25 μg/μl) + S-NLCs (25 mg/kg) + I/R, **f**: The percentage of brain infarcted area with respect to the total area. Data are represented as mean ± SEM (n = 6). ^***^ P < 0.001 vs. sham, ^#^ P < 0.01 vs. I/R group. (TTC: 2,3,5-triphenyltetrazolium chloride, I/R: ischemia/reperfusion, LY: LY294002, S-NLCs: sesamol-loaded nanostructured lipid carriers)

TTC staining revealed the significantly increased infarcted areas in I/R group [Fig. 8b and f, *P*<0.001 vs. sham group (Fig. 8a and f)]. FA even at the highest dose tested (25 mg/kg) failed to prevent I/R-induced brain infarction (Fig. 8d and f, *P*>0.05 vs. I/R group), while, FA-NLCs (25 mg/kg) showed protective effect (Fig. 8c and f, *P*<0.01 vs. I/R group). Pre-treatment with LY294002 (25 μg/μl) abolished the protective effect of FA-NLCs (Fig. 8e and f, *P*>0.05 vs. I/R group). The lower doses of LY294002 were ineffective and the antagonist showed no effect by itself (not shown).

#### The effects of sesamol or S-NLCs on I/R-induced neuronal damage in the hippocampal CA1 region

**Fig. 9:**
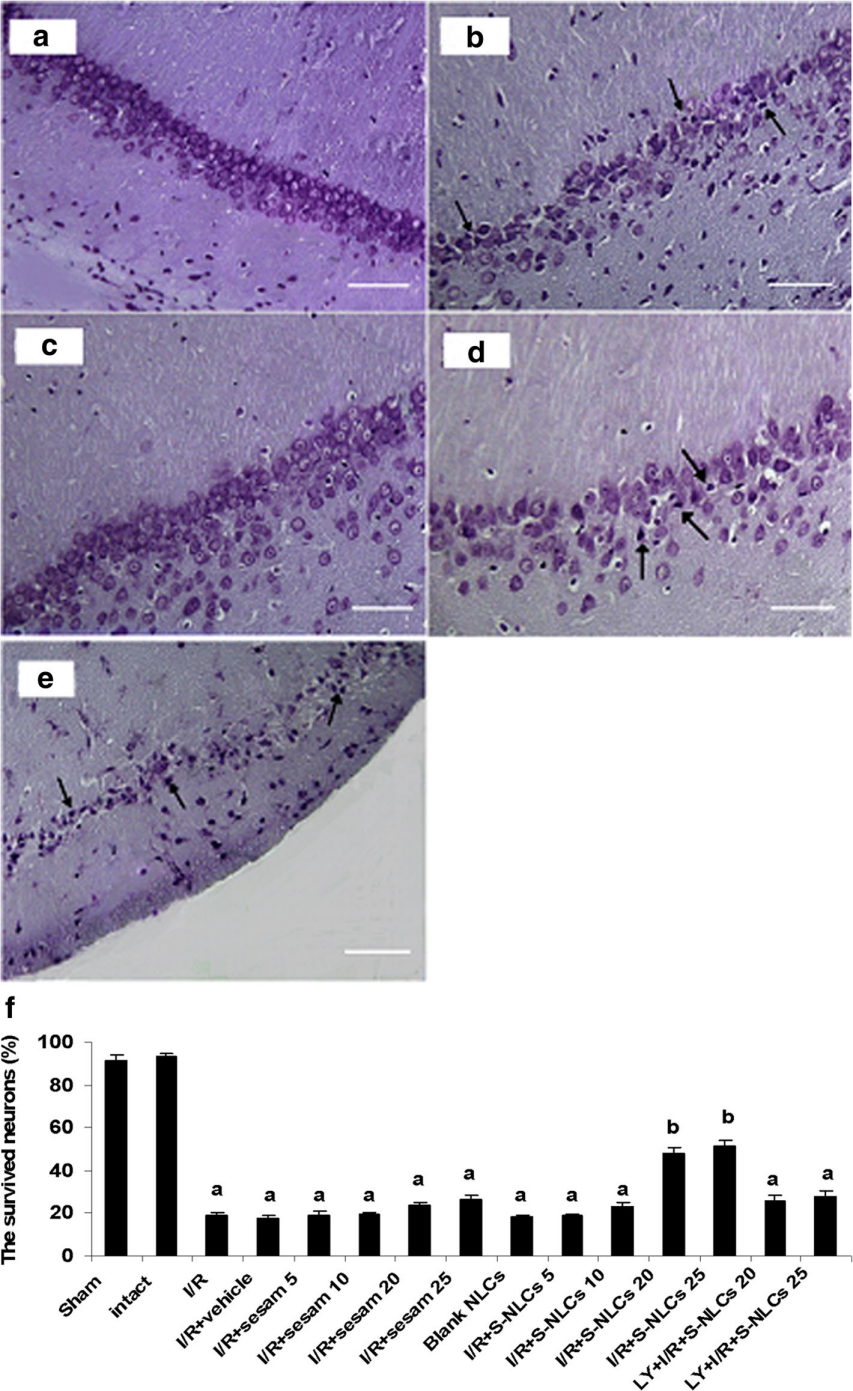
Representative photomicrographs of hippocampal slices and quantitative analysis of the survived neurons in CA1 sub-regions. **a**: The viable round cells with distinct nucleus and nucleolus in sham-operated group, **b**: Dark-stained cells with dysmorphic or shrunken shape (black arrows) due to 10-min ischemia followed by 72 h reperfusion, **c**: Treatment with S-NLCs (containing 25 mg/kg of sesamol) attenuated I/R-induced neuronal damage, **d**: Free sesamol (25 mg/kg) failed to effectively prevent I/R-induced neuronal injury, **e**: Pre-treatment with LY294002 (25 μg/μl) abolished the ameliorative effects of S-NLCs (25 mg/kg), (scale bars: 50 μm). **f**: S-NLCs (20 and 25 mg/kg), but not sesamol, significantly increased the survived neurons in the hippocampal CA1 region. Pre-treatment with LY294002 (25 μg/μl) prevented the neuroprotective effects of S-NLCs. Data are expressed as mean ± SEM (n = 6). ^a^ P < 0.001 vs. sham or intact group, ^b^ P < 0.001 vs. sham, intact, I/R or I/R + vehicle groups. (I/R: ischemia/reperfusion, LY: LY294002, S-NLCs: sesamol-loaded nanostructured lipid carriers)

Based on the histological evaluation, global cerebral ischemia induced the neuronal damage in the hippocampal CA1 region as revealed by the formation of dark-stained cells with dysmorphic shape (Fig. 9b) and less survived neurons (Fig. 9f, *P*<0.001 vs. sham or intact group) as compared to the sham group with closely arranged round cells including the distinct nucleus and nucleolus (Fig. 9a). Treatment with S-NLCs (25 mg/kg) improved the cell morphology (Fig. 9c) and significantly increased the number of survived cells (Fig. 9f, *P*<0.001 vs. I/R or I/R + vehicle group), while, free sesamol even at the highest dose tested (25 mg/kg) was ineffective against I/R-induced neuronal damage (Figs 9d and f, *P*>0.05). Pre-treatment with LY294002 (25 μg/μl) abolished the neuroprotective effect of S-NLCs (Figs. 9e and f, *P*>0.05 vs. I/R group or I/R + vehicle group), while, pretreatment with lower doses of LY294002 showed no effect (not shown). This PI3K antagonist showed no effect by itself (not shown).

#### The effects of sesamol or S-NLCs on I/R-induced oxidative stress in the hippocampus

As shown in Table 5, I/R led to the significant elevation of MDA (*P* < 0.01) and reduction of GSH (*P* < 0.001), SOD (*P* < 0.001), and CAT (*P* < 0.001) as compared to the sham or intact group. This, was prevented by 20 and 25 mg/kg of S-NLCs (MDA: *P* < 0.01; GSH: *P* < 0.05, *P* < 0.01; SOD: *P* < 0.01, *P* < 0.001; and CAT: *P* < 0.01 vs. I/R or I/R + vehicle.), while, free sesamol failed to prevent I/R-induced oxidative stress at any dose tested (*P* > 0.05 vs. I/R or I/R + vehicle). Pre-treatment with LY294002 (25 μg/μl) reversed the protective effect of S-NLCs against I/R-induced oxidative stress (*P* > 0.05 vs. I/R group or I/R + vehicle group), however, the lower doses of LY294002 were ineffective (not shown). LY294002 showed no effect by itself (not shown).

**Table 5:** Effects of sesamol or S-NLCs on the biochemical parameters in hippocampus following the global ischemia

Groups	MDA (nM/mg protein)	GSH (nM/mg protein)	SOD (U/mg protein)	CAT (U/mg protein)
Sham Intact I/R I/R + vehicle I/R + sesamol (5 mg/kg) I/R + sesamol (10 mg/kg) I/R + sesamol (20 mg/kg) I/R + sesamol (25 mg/kg) I/R + blank NLCs I/R + S-NLCs (5 mg/kg) I/R + S-NLCs (10 mg/kg) I/R + S-NLCs (20 mg/kg) I/R + S-NLCs (25 mg/kg) LY + I/R + S-NLCs (20 mg/kg) LY + I/R + S-NLCs (25 mg/kg)	0.92 ± 0.06 0.87 ± 0.09 2.12 ± 0.21^**^ 2.16 ± 0.19^**^ 2.09 ± 0.20^**^ 2.23 ± 0.18^**^ 1.93 ± 0.20^*^ 1.84 ± 0.19^*^ 2.03 ± 0.34^*^ 2.14 ±0.24^**^ 1.95 ± 0.15^*^ 1.29 ±0.15^Ω^ 1.08 ±0.12^#^ 2.13 ±0.16^**^ 1.99 ±0.22^*^	1.98 ± 0.09 2.02 ± 0.18 0.96 ± 0.22^***^ 0.91 ± 0.15^***^ 0.98 ± 0.17^**^ 0.87 ± 0.15^***^ 1.04 ± 0.06^**^ 1.09 ± 0.13^**^ 1.01 ± 0.19^**^ 0.98 ± 0.15^**^ 0.93 ± 0.24^***^ 1.63 ± 0.09^Ұ^ 1.81 ± 0.07^∑^ 0.93 ± 0.13^***^ 1.07 ± 0.09^**^	5.77 ± 0.25 5.83 ± 0.19 3.31 ± 0.13^***^ 3.24 ± 0.21^***^ 3.13 ± 0.15^***^ 3.68 ± 0.25^***^ 3.60 ±0.37^***^ 3.79 ±0.19^***^ 3.31 ± 0.11^***^ 3.19 ± 0.20^***^ 2.77 ± 0.23^***^ 4.68 ± 0.19^Δ^ 4.97 ± 0.14^£^ 3.07 ± 0.24^**^ 3.16 ± 0.11^***^	4.69 ± 0.32 4.58 ± 0.54 2.49 ± 0.17^***^ 2.55 ± 0.22^***^ 2.63 ± 0.24^***^ 2.68 ± 0.18^***^ 2.77 ± 0.12^***^ 2.61 ± 0.15^***^ 2.83 ± 0.25^***^ 2.80 ± 0.16^***^ 3.13 ± 0.14^**^ 4.06 ± 0.29^Φ^ 4.29 ± 0.12^€^ 2.66 ± 0.19^***^ 2.37 ± 0.98^***^

S-NLCs, but not free sesamol, significantly attenuated the oxidative stress induced by the global ischemia. Data are presented as mean ± SEM (*n* = 6)

(*I/R* ischemia/reperfusion, *NLCs* nanostructured lipid carriers, *S-NLCs* sesamol-loaded NLCs, *MDA* malondialdehyde, *GSH* reduced glutathione, *SOD* superoxide dismutase, *CAT* catalase)

^*^P < 0.05, ^**^ *P* < 0.01, ^***^ *P* < 0.001 vs. sham or intact, ^Ұ^P < 0.05, ^Φ, Δ, Ω, #, ∑, €^P < 0.01, ^£^P < 0.001 vs. I/R or I/R + vehicle

## Discussion

Cerebral stroke has remained a major healthcare challenge. Recombinant tissue plasminogen activator (r-tPA) remains the sole medication, however, it should be administered in a short time after onset of symptoms [1]. Over the last decade, phenolic compounds such as sesamol have been the focus of intense research due to their antioxidant and neuroprotective properties, however, poor solubility or rapid metabolism may negatively affect their effectiveness [12, 13, 58–61]. In the present study, we have prepared S-NLCs as the nanoreserviors to provide longer-lasting therapeutic effects for sesamol in both in vitro and in vivo models of ischemic stroke. S-NLCs showed high stability profile during 6-month follow up (Table 2) that might be due to the binary mixture of solid and liquid lipids which elevates the imperfection and minimizes the drug expulsion. The homogeneous S-NLCs with narrow particle size distribution and suitable ZP (Table 1), preserved their uniformity and spherical shape during the storage (Fig. 1) indicating the suitability of materials and preparation technique. In contrast to SLNs in which the aggregation or perikinetic flocculation may occur during the storage [27–29], the particles in highly-concentrated NLCs formed a network (Fig. 1b) that may not result in the collision of particles during the long-term storage.

Thermal behaviours of bulk materials, blank NLCs, and S-NLCs were determined by DSC (Fig. 2). The disappearance of sharp endothermic peak of pure sesamol in DSC thermogram of S-NLCs suggests that sesamol is distributed in an amorphous status. Furthermore, the melting peaks of blank- or S-NLCs shifted to the lower temperature indicating that the bulk materials have been transformed into the nanoparticulate forms. Small particle size and enhanced surface of nanopartiocles may also result in the depression of melting point [62]. S-NLCs showed lower melting point as compared to the blank NLCs that might be due to the incorporation of sesamol into the lipid matrix.

A hyperbolic trend in the release profile (Fig. 3) indicates the controlled-release pattern of sesamol from NLCs which may be due to the partitioning of sesamol between the lipid and aqueous phases and interactions between the surfactant-lipid or sesamol-lipid molecules. The prolonged release may also be attributed to the diffusion of sesamol from the lipid core of NLCs. This kind of release pattern by providing a constant concentration of sesamol for longer time period might be of therapeutic value.

Since the cell internalization of nanoparticles is highly influenced by their size and surface properties, we selected S-NLCs7 as the optimal formulation due to the suitable EE%, DL%, and particle size (Table 1). These types of nanoparticles with particle diameter less than 100 nm and large surface area have shown unique biological effects such as the ability to bypass the reticulo- endothelial system. Furthermore, the probability of destruction or phagocytosis is minimized [27–29] that may result in the enhanced retention time and efficiency. In this respect, these nanoparticles have been suggested as suitable carriers for effective drug delivery into the brain [27]. Regarding the bioactivity of S-NLCs in an in vitro model of ischemic stroke, MTT assay revealed that S-NLCs, but not free sesamol, exert dose-dependent protective effects in longer exposure to OGD/R (Fig. 4) indicating the ability of this nanocarrier to provide a sustained concentration of sesamol. The cellular damage was also evaluated by measuring an indicator of cell toxicity, LDH, the cytoplasmic enzyme which is rapidly released into the cell culture medium following the damage of cell plasma membrane. In general, increased LDH activity has been recognized as a marker for the ischemic processes or cell death [39]. As shown in Fig. 5, sesamol solution failed to prevent the enhancement of LDH due to 8-h OGD, while, S-NLCs showed efficiency at both 1- and 8-h OGD indicating the ability of this nanocompound to protect sesamol against the rapid metabolism leading to the prolonged protective effects.

Unlike the free sesamol, S-NLCs protected PC12 cells, a model of neuron-like cells, against the morphological alterations due to 8-h OGD (Fig. 6) indicating the prolonged therapeutic effect of S-NLCs. As shown in Table 3, S-NLCs, but not sesamol, dose-dependently suppressed 8-h OGD-induced oxidative stress as revealed by the ability of this nanocarrier to reduce the production of MDA, an indicator of free radical generation, oxidative stress, and tissue injury [63], and enhance the content of GSH, a free radical scavenger and an essential component of cellular defence mechanism against the oxidative stress [64], and activities of SOD, an essential enzyme for the removal of superoxide radicals and protecting the cells against the oxidative injury [65], and CAT which is responsible for degradation of H_2_O_2_ and protects the cells against the oxidative stress [66]. These findings indicate the ability of NLCs to provide longer-lasting effects for sesamol. Based on the protective effects of sesamol against the neuronal injury [6–8, 11–13, 67], the extended activity of this phenolic compound might be of therapeutic significance against the cellular dysfunction in the acute or chronic forms of neural injury.

Besides the application of the visualization technique and brain distribution study (Fig. 7), evaluation of the bioactivity of S-NLCs in an in vivo model of ischemic strike confirmed the successful delivery of these nanoparticles into the rat brain. As shown in Table 4 and Fig. 8, S-NLCs effectively prevented I/R-induced neurobehavioral deficits. S-NLCs, but not free sesamol, significantly attenuated I/R-induced histopathological alterations and cell loss in the CA1 hippocampal region (Fig. 9) which is one of the most vulnerable brain regions in the cerebral ischemia [68]. Even after a few minutes of global brain ischemia, the pyramidal cells of CA1 sub-region become irreversibly damaged [68, 69]. Therefore, S-NLCs by providing sustained concentrations of sesamol might be of therapeutic value in I/R-induced neuronal injury.

Because of its high rate of oxidative metabolic activity, brain is one of the most vulnerable tissues to the oxidative stress [69]. Antioxidant enzymes including the SOD and CAT by eliminating the free radicals protect the brain against the ischemic insult [70]. Furthermore, GSH which is involved in the maintenance of cell integrity, serves as a primary defensive agent against the oxidative stress in the brain [7, 65]. In this respect, S-NLCs by potentiating the antioxidant defense system and attenuation of I/R-induced oxidative stress in the hippocampal region (Table 5) may exert promising neuroprotective effects. Indeed, NLC-based formulations are promising therapeutic candidates against a variety of pathological conditions including the ophthalmic disorders [71, 72].

Sesamol even at the highest dose tested failed to significantly affect I/R-induced neurobehavioral deficits and oxidative stress (Figs 8 and 9, Tables 4 and 5) that might be due to the rapid metabolism and/or limited number of injections leading to the subtherapeutic concentrations in brain. In previous reports, sesamol has shown efficiency following 14–21 daily injections [9, 11, 12], while, in the present study, S-NLCs following the limited number of injections exhibited therapeutic effects indicating the enhanced bioavailability and efficiency of sesamol following its incorporation into the NLCs. Application of LY294002 abrogated the protective effects of S-NLCs (Figs. 4-6, 8 and 9, Tables 3-5) indicating that S-NLCs, like the classical neuroprotective agents, are able to recruit PI3K signalling pathway.

## Conclusions

NLCs serve as promising carriers for sesamol, a phenolic compound with a wide spectrum of pharmacological activities. S-NLCs by improving the pharmacological profile of sesamol provide longer-lasting therapeutic effects in both in vitro and in vivo models of stroke. This nanoformulation through the activation of PI3K pathway might be a suitable controlled release drug carrier system against the ischemic injuries or other neurodegenerative pathologies.

## Abbreviations

CAT: Catalase
DL: Drug loading
DMSO: Dimethyl sulfoxide
DSC: Differential scanning calorimetry
EE: Entrapment efficiency
GSH: Reduced glutathione
HPLC: High-performance liquid chromatography
I/R: Ischemia/reperfusion
LDH: Lactate dehydrogenase
MDA: Malondialdehyde
MTT: 3-[4,5-dimethylthiazol-2-yl]-2,5-diphenyl tetrazolium bromide
MWM: Morris water-maze
OGD: Oxygen-glucose deprivation
PBS: Phosphate buffered saline
PDI: Polydispersity index
PI3K: Phosphatidylinositol 3-kinase
S-NLCs: Sesamol-loaded nanostructured lipid carriers
SOD: Superoxide dismutase
STPAT: Step-through passive avoidance test
TBA: Thiobarbituric acid
TTC: 2,3,5-triphenyltetrazolium chloride
ZP: Zeta potential
